# Agent-based modeling and life cycle dynamics of COVID-19-related online collective actions

**DOI:** 10.1007/s40747-021-00595-4

**Published:** 2021-12-17

**Authors:** Gang Zhang, Hao Li, Rong He, Peng Lu

**Affiliations:** 1 School of Economics and Management, Shaanxi University of Science and Technology, Xi’an, China; 2 School of Economics and Management, Xinjiang University, Xinjiang, China

**Keywords:** COVID-19, Online collective actions, Agent-Based Modeling (ABM), Substitution effects, Attention shift and attention allocation

## Abstract

The outbreak of COVID-19 has greatly threatened global public health and produced social problems, which includes relative online collective actions. Based on the life cycle law, focusing on the life cycle process of COVID-19 online collective actions, we carried out both macro-level analysis (big data mining) and micro-level behaviors (Agent-Based Modeling) on pandemic-related online collective actions. We collected 138 related online events with macro-level big data characteristics, and used Agent-Based Modeling to capture micro-level individual behaviors of netizens. We set two kinds of movable agents, Hots (events) and Netizens (individuals), which behave smartly and autonomously. Based on multiple simulations and parametric traversal, we obtained the optimal parameter solution. Under the optimal solutions, we repeated simulations by ten times, and took the mean values as robust outcomes. Simulation outcomes well match the real big data of life cycle trends, and validity and robustness can be achieved. According to multiple criteria (spans, peaks, ratios, and distributions), the fitness between simulations and real big data has been substantially supported. Therefore, our Agent-Based Modeling well grasps the micro-level mechanisms of real-world individuals (netizens), based on which we can predict individual behaviors of netizens and big data trends of specific online events. Based on our model, it is feasible to model, calculate, and even predict evolutionary dynamics and life cycles trends of online collective actions. It facilitates public administrations and social governance.

## Introduction

The COVID-19 was first discovered in December, 2019, and declared a global pandemic by WHO on March 11st of 2020 [1]. It greatly threatens global public health [2], with 178,837,204 infected and 3,880,450 died worldwide, by June 23th of 2021 [3]. Revealing characteristics and developing treatments become a core task for global scientists and studies such as pathology analysis [4, 5], patient treatments and influences [6–16] have been done. For example, Srivastava et al. analyzed non-chemical signals of biological elements, a unique biophysical feature of COVID-19 [4]; Raza et al. proposed ‘Primed’ Mesenchymal Stem Cells (MSCs) as a therapeutic alternative [5]; Ragnesola et al. evaluated 63 samples of concentrated plasma (CP) from New York Blood Center (NYBC) using side flow analysis (LFA) platform [6]; and Prada et al. studied COVID-19 infection risk through high-dose immunoglobulin pulse therapy [7]. Attention has also been paid to humanity aspects under the pandemic, such as societal impacts [10, 11], information dissemination [12], health care [13, 14], and technology[15–17]. For instance, Camporesi et al. analyzed several potential iatrogenic causes of the detrimental effects on children and highlighted the risks under sudden changes of clinical practice [8]; Antonelli et al. found that SPA facilities can be proper settings of respiratory rehabilitations, for those post-pandemic patients [9]; Chowdhry et al. developed guidelines that can mitigate its impact on health care [10]; Coltrain et al. found the staggering patterns of inequitable mortality by race and ethnicity for US big cities [11]; Davalbhakta et al. performed a quality assessment of multiple mobile applications (APPs) to evaluate their effects [12]; Speth et al. believed that smokers might be at a higher risk [13]. Khosla et al. lead a team and rolled out two variants of telemedicine solutions for use by national populace of India, mitigating the Impact of Covid-19 [14]. Brohi et al. have unveiled five state-of-the-art technologies, which includes Artificial Intelligence (AI), 3D Printing Technology (3DPT), Big Data Analytics (BDA), High Performance Computing (HPC) and Telecommunication Technology (TT), and their remarkable applications that can be used to mitigate and eliminate the problems of COVID-19 [15]. Mittal et al. designed a framework model with the integration of both cloud and artificial intelligence technologies to mitigate the impact of COVID-19 on seafarers’ mental health [16]. Dogan et al. reviewed 264 studies on AI/ML approaches against COVID-19. They think that their study can be a key element for epidemic and transmission prediction, diagnosis and detection, and drug/vaccine development [17].

The pandemic has also brought chaos and impacts on the cyberspace, but we have not witnessed enough studies on pandemic-related online events. Virtual society was generated, because the rapid development of ICT has changed human behavior patterns, narrowed boundaries between countries [18]. Online collective actions in this virtual society reflect both virtual and real societal features, and have brought great impacts on global public security and social governance [19]. Besides infection and death, the pandemic threatens public safety and has negative psychological effects as well [20]. Zhong performed text analysis, sentiment analysis, and correlation analysis, to investigate and reveal what netizens thinks, feels, and concerns, under this pandemic [21]; Qazi et al. evaluated how online public opinions shape situational awareness of individuals for adopting health-protective behaviors [22]; Wang et al. used the big data of social media (Twitter) posts to explore public opinions toward COVID-19 [23]; Chen et al. considered the internal characteristics and external information characteristics of online public opinion, built a model, combined with a case to study the formation of COVID-19 online public opinion [24]; Zhuang et al. propose the LDA-ARMA deep neural network for dynamic presentation of COVID-19 public-opinion events [25]. Lee et al. analyzed the perceptions and emotions of Korean and Japanese citizens regarding COVID-19, and then suggested focusing policies toward economic stability is essential [26]. All these studies focus on the attention of Internet users (netizens) with a few case studies, other than the overall trends of related online collective actions. Here, we focus on pandemic-related online collective actions, especially evolutionary dynamics of the life cycles. This work helps to stabilize social emotions, public feelings, and social psychology under this pandemic. Online collective action is the macro-level emergence, induced by aggregated micro-level behaviors of participants (individuals). The life cycle phenomena have been investigated in many fields, such as industrial production [27], biochemistry [28], life health [29], and software application [30]. The life cycle pattern is a universal law in multiple fields, and it can be also applied to emergency event cases and crisis management. In 1985, the National Governors Association (NGA) first put forward the Four-Stage Theory on crisis management [31]. Burkholder et al. proposed the well-known three-stage model for the life cycle stages of public opinions on emergencies [32]. However, less attention has been paid to overall trends and evolutionary dynamics, although the life cycle pattern is universal.

As a typical method of social computing, Agent-Based Modeling can be used to explore the autonomous actions of individual interactions. In other words, it can use micro-level behavior rules to study and predict macroscopic emergent complex phenomenon [33]. Intelligent agents behave under a certain environment, with their own behavior rules and learning abilities [34]. According to the environment, the agents can perceive the environment, adjust their own states, decisions, and behaviors, and even influence others. After a decision has been made, the agent can take actions to achieve its own goals or the goals given by the environment [35]. Agent-Based Modeling has two advantages: (a) it connects macro- and micro-level researches. It explores macro-level phenomena from micro-level perspectives. Under simple micro-level behavior rules, it predicts complex macro-level phenomena, which is the core principle of complexity and system sciences and is effective to study the emergent phenomena under interactions of agents (netizens). Statistical models can discover the knowledge through static data, and simulations can find related mechanisms under the dynamic processes. According to ABM simulations, the macro-scale model is caused by micro-level behaviors, guided by the idea that the macro-level phenomenon is produced by the dynamic process of micro-level behaviors [36]. This theoretically associates macro- and micro-level studies; (b) it can be applied on interdisciplinary studies. ABM is widely used in various disciplines. For instances, Carley and Newell explored the coevolution of social networks and culture using ABM [37]; Axtellet et al. studied some social phenomena, such as seasonal migration, pollution, reproduction, wars, epidemics, diseases, and even cultural transmission, using ABM simulations [38]; Deissenberget et al. described the general structure of the economic model, by establishing the agent model of labor market [39]; Griffié et al. used it to study the molecular aggregation on plasma membrane [40]; Liao et al. applied a DEB-ABM to the Japanese anchovy Engraulis japonicas, captured energy acquisition and allocation throughout the anchovy life cycle, and predicted how individual-level processes affect energy dynamics at higher levels of biological organization [41]. Matias et al. simulate the behavior of the Douro Region farms about the changes in the price of labor, through the method of ABM [42]. Here, we use Agent-Based Modeling to explore the dynamics of life cycles.

This paper proposes the Agent-Based Modeling of the life cycle dynamics, and aims to reveal individual behaviors and evolutionary dynamics of pandemic-related online collective actions. The macro-level life cycle pattern is jointly determined by both positive and negative mechanisms. The positive mechanism of online collective actions is obvious, and the key to understand and solve the whole life cycle pattern is the negative mechanism. We aim to answer three specific questions: (a) When does the negative or restraint mechanism work for single event? This negative mechanism does not merely exist at the decline stage (after the peak), but also within the whole life cycle process. For single or individual online events, the life cycle pattern is robust and persistent. For multiple events, their specific life cycle shapes or curves may be slightly affected and influenced by other concurrent events; (b) What are the interactions and relationships within multiple life cycle trends (events). For multiple (coexisting) events, there ought to be some interactions (substitution effects) between them in cyberspace. Based on the observed big data, newly emerging events (online collective action) often lead to accelerated declines of current existing events. It is highly possible that there are some alternative or substitution effects, because the life cycle trends can be shaped by other factors, and they may influence each other; and (c) What is the ultimate or overall restrictive mechanism? Both objectively or subjectively, all online collective actions are seeking or competing for the online attention flows from the people online (netizens). Online attention flows of netizens take on the behaviors of online Internet browsing, webpage clicks, and social media posting & sharing. The critical (key) feature is that attention always flows, switches among different online events. Hence, the dynamic distribution of attention flows determines the whole life cycle process, based on which we build our Agent-Based Modeling. According to the outcomes of big data mining, we seek the optimal solutions of the simulations to best match the real big data outcomes. Combining both Agent-Based Modeling and big data mining methods, we are to improve both validity and robustness of pandemic-related online events.

Research designs are as follows: Based on life cycle phenomenon and attention–allocation mechanism, we collect and analyze real big data of COVID-19-related online collective actions, using ABM method to model them dynamically, and traverse all parameter values to find the optimal solution, which best matches the exact number of real big data events of COVID-19. After the optimal solution is obtained, we repeated the simulation ten times, and take the mean values as the robust outcomes. Hence, multi-dimension matching degrees between simulations and real big data have been calculated and visualized to check both validity and robustness of our model. Multi-dimensional matching includes event number matching, life cycle matching, duration matching, peak matching, and substitution-effects matching and so on. The matching results show that our model is stable and robust. Therefore, our proposed model is feasible to predict online collective behavior and dynamic evolutionary trends of online public-opinion events for possible artificial interventions and effect evaluations. This model seized the core behavior mechanism of netizens, and revealed the interactions and substitution mechanism among multiple events. This model can also be used to guide the real online collective actions, such as predicting the trends, durations, and effects of their life cycle processes.

## Existing studies and perspectives

### Positive or facilitating mechanism

Regarding the growth mechanism, commonly cited motivative factors are: (a) Online openness and anonymity of the Internet. The openness and anonymity of the Internet are commonly believed to have facilitated the outbreaks of online events. For the openness, almost all people worldwide have access to the (mobile) Internet. For the anonymity of the Internet, there are some disputes, but people all believe that it facilitates the outbreaks of more online events. Supporters believe that the openness makes it assured that governments cannot spy on citizens and thus guarantees the rights of privacy and free speech [43]. Opponents believe that it brings more misleading information to society [44]. Now, the Internet is still anonymous, which generates more events. Compared to face-to-face communications (interactions), people (netizens) bear less human sympathy, morality, and legal pressures online. Internet language style is more random and primitive. The anonymity and openness provide major channels for the release or expression of social contradictions and social grumpiness, which causes more online events; (b) the cost of online participation is reduced with little space & time restrictions. The cost of communication has been greatly reduced, and the development of Internet technology has broken space & time boundary of information dissemination. The “Network Society” constructs a new social space–time [45], and the costs of individual expressions and public participations have been greatly reduced. It is normal and free for people to speak, support, and even criticize others online, without being punished. Under the big data era, the mobile Internet aggravates this process. Mobilized by social media Apps [46], unfamiliar people can form strong and lasting social relations. People can participate at public events online at any time and from anywhere, which promotes the formation and outbreak of online collective actions; and (c) the group polarizations within online collective actions. Group polarization refers to collective attitude, which can be formed after group discussion and individual interactions. It tends to be more extreme after group discussions, compared with the average attitude of group members or individuals. Intergroup attitudes and emotions can stimulate and regulate intergroup attitudes and behavior changes [47]. Compared with face-to-face communications in reality, the polarizations of network groups are obviously more extreme and polarized [48].

### Negative or constraint mechanism

The life cycle pattern is universal for online collective actions. This pattern is jointly formed by both positive and negative mechanisms. The negative one refers to attention distributions, such as the substitution and restriction effects, and is the core to understand the life cycles. The attention distribution mechanism has the following features: (a) evolutionary psychology principle of the attention distribution. Attention is the basic thinking pathway for the biological world (human beings and other animals). For human beings, attention should be distributed to satisfy their cognitive needs [49]. To avoid unnecessary energy waste and improve the efficiency, animals and human beings should focus on key objects and ignore irrelevant objects temporarily; (b) interdisciplinary mechanism of the attention distribution. Related researches are interdisciplinary, which involves disciplines like psychology [50], sociology [51], economics [52], and management [53]. Attention is scarce, and it is impossible to receive too many signals, when people are making decisions [54]. Due to the limitations of energy and ability, it is necessary for people to rank things and make priorities; and (c) total attention restriction and netizen attention distributions. The limitation of attentions is the deeply rooted mechanism. Under embedded social structure and fragmented personal time [55, 56], netizens merely focus on few news or events. Dominated by this, the time, energy, clicks, responses, comments, social networking, and (dis)likes are all limited or scared resources. Thus, netizens allocate their attentions to different online events, which generates multiple coexisting life cycles. In Table 1, we provide both positive and negative mechanisms of online collective actions.

**Table 1:** Positive and negative mechanisms of life cycles

	Single life cycles	Multiple life cycles
Positive or facilitating	Openness Anonymity Polarizations Reduced cost	Openness Anonymity Polarizations Reduced cost
Negative/constraint	Recession Substitution Attention shift Attention allocation	Recession Substitution Attention shift Attention allocation

### Combined positive–negative mechanism

Because of its openness, anonymity, and low cost, the Internet provides more channels for individuals to express and participate online. Based on the limited attention of netizens, we have the substitution effect in the multi-event system. For single event, the life cycle is largely determined by both positive and negative mechanisms. For coexisting events, the life cycles are also influenced by the effects of substitution and attention shift, which is ultimately determined by the limited amount of attention. Determined by this combined mechanism, it seems that: (a) online collective actions take places normally and randomly. On a daily basis, they are continuously triggered by individual participation and group behaviors, such as (dis)likes, comments, interactions, sharing, re-twittering, forwarding, etc., which generates the life cycle pattern. There are a certain number of hot events in online events with only some hot events each day; (b) the life cycle pattern is robust but heterogeneous. For specific life cycles, they are also infected by related factors, which leads to heterogeneity. For example, they can be agenda setting and political evaluation [57], or people’s demands are behind online group events [58]. These factors merely influence the big data trends and shapes, but cannot change the law of life cycle pattern; and (c) the life cycle is comprehensively determined by the effects of positive, recession, substitution, and attention allocations. The life cycle of a single event is mainly determined by the facilitating (positive) and recession (negative) mechanisms. For multiple events, the substitution mechanism also works. For both single event and multiple events, the entire online collective actions are restricted by total attention. The total attention paid to specific events determines the life cycle features, such as durations and peaks. For pandemic-related online events, they are also applicable. Based on these mechanisms, we build the Agent-Based Modeling, whose simulations should match real big data of pandemic-related online events.

## Big data mining of target life cycles

**Fig. 1:**
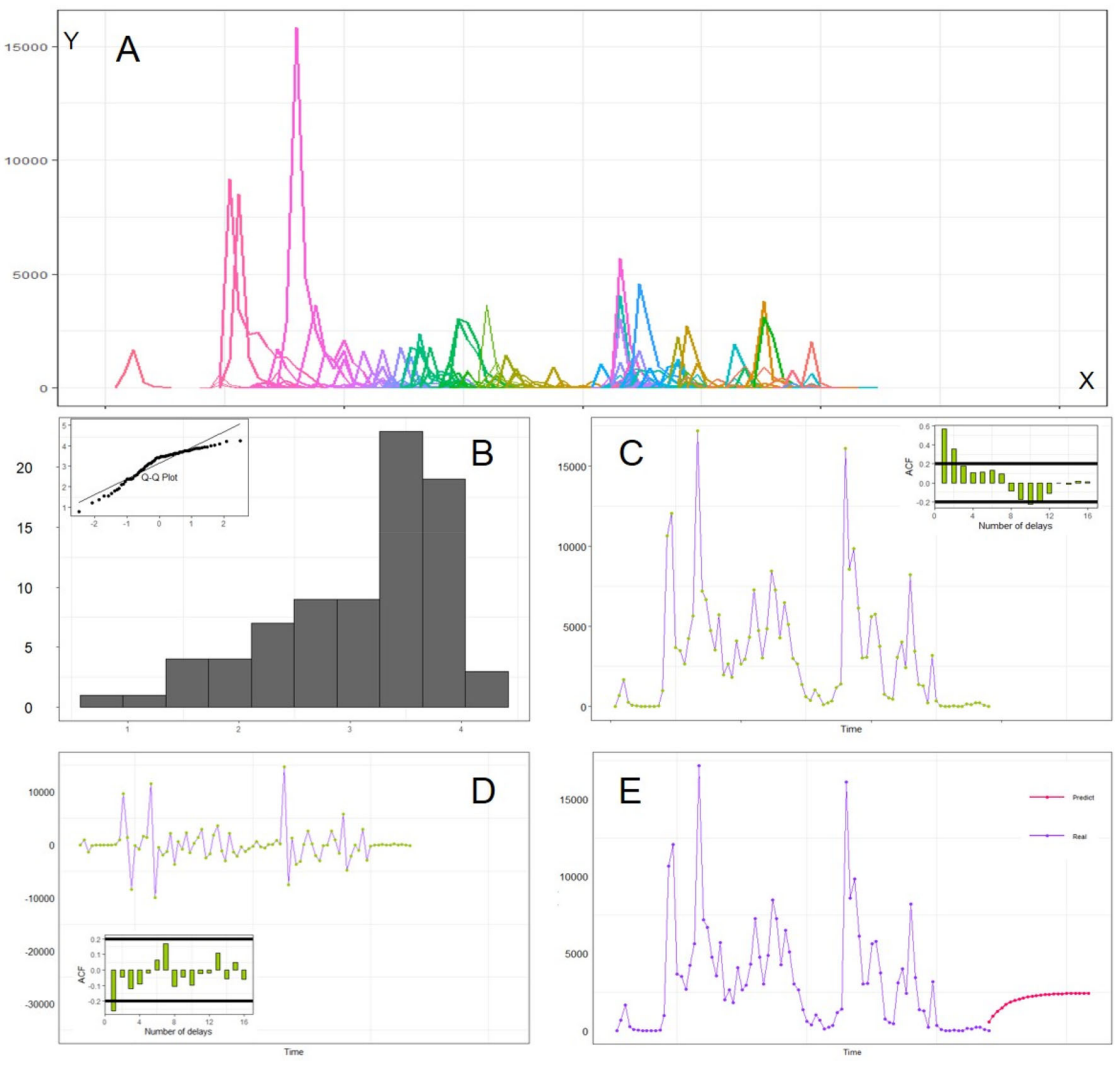
The characteristics and big data trends of COVID-19 online collective actions. Panel **A** provides the big data trends (curves) of 138 related events. Panel **B** visualizes and checks the distributions of durations or lifespans (*N* = 138). Panels **C** & **D** execute time-serial analysis of real big data. We predict time-serial values in Panel **E**. It indicates the limitation of online attentions and attention-shift mechanism

For Agent-Based Modeling, real online events are needed for real targets of our simulations. In this work, we have collected big data trends of life cycles for the pandemic-related online events as the real targets: (a) data sources and big data analysis of real event. We use the big data platform (zhiweidata.com) to extract the life cycle curves of COVID-19 events. The zhiweidata.com is a free data-collection platform of online collective action events from open sources. The data are mostly from mainstream and official online medias in China and are comprehensively trustworthy. This is why, we use them. For other platforms, the pattern is similar. Although the peak heights of the online events of other platforms may be different, it has little impacts on the results, because the core indicators (peaks, durations and trends) of the life cycles are the same. For these life cycle curves, the *Y*-axis is the volume of posts; the *X*-axis refers to the date of posts. This platform mainly presents events with a high level of online dissemination within a short period of time which have aroused hot discussions online. We took the events happened within 3 months, from January 19th to April 21st, 2020, with *N* = 138 pandemic-related events; (b) big data trends of life cycles. The number of COVID-19-related events is large compared with this short period of time, and there are as many as 138 events in 3 months, roughly one event each day. Figure 1A shows the life cycle tendency of the 138 real target events we plot. It seems that events frequently break out within a short time, and the substitution effect is therefore obvious. The dynamic amount of the online participation captures the life cycle trends. The higher peak (with more posts) shows more public participation and more online attention. The durations of the events are heterogeneous: there is only one event which lasted for 41 days, and the rest usually lasted for 2–10 days; (c) substitution effects. Online events usually overlap each other, and have more than two events alive at each day. As well, we have various peak heights, which means that the difference exists. There are intersections among the curves. One event often occurs at the time other events are occurring but in a chronological order, which supports the substitution effects. It also confirms that the total volume of the netizens’ attention is limited. There are also time differences (gaps) between peaks, which indicates that there is substitution effects between coexisting events, although some of them are not strongly related; (d) attention observation mechanism. The attention volume of netizen is (peak) limited with characteristics of randomness. Hence, total attention paid to this multi-event system fluctuates around certain average levels. Figure 1B visualizes the distribution of total attention (daily) in logarithm, which is close to the normal distribution. Hence, the certainty (mean) and randomness (SD) can be held; and (e) time-serial analysis and prediction. In Fig. 1C, the autocorrelation chart shrinks from the third-order lag column into a stable interval, and then breaks through the stable interval again on negative terms. Therefore, the original data are nonstationary. The seasonal factor structure is the set {0.677, 0.65, 1.031, 1.354, 1.645, 0.871, 0.773}, from Sunday to Saturday. Higher seasonal factors are Tuesday (1.031), Wednesday (1.354), and Thursday (1.645). As in Fig. 1D, the stationary time-serial trend is obtained after we take the first-order difference processing, which can pass the inspection of the Ljung–Box randomness test. The *H*_0_ is time-serial uncorrelated, and *H*_1_ is serial correlation. Because *P* value = 0.24 > 0.05, the *H*_0_ should be accepted. Hence, the time-serial trend is uncorrelated (white noise), and the data information can be better expressed by this model. Based on stationary serial prediction in Fig. 1E, the number of daily postings fluctuates approximately around a constant, within a small range of variances. Based on the predicted trend (in red), it seems that the volume of total attention paid by netizens is a fixed constant, which reinforces the limitation of online attentions and the attention-shift mechanism.

## Agent-based modeling and simulations

Based on related mechanisms of individual and group behaviors, we build an Agent-Based Modeling, whose simulations have matched the big data trends, as well as the time-serial analysis of the (*N* = 138) real target events.

### Paradigms of online collective action studies

There are three paradigms in online collective action studies: (a) system dynamics simulations. Focusing on systemic structures, it takes selectivity, self-discipline, and non-linearity into considerations [59]. According to Sterman, it can build formal simulations of complex systems to help design more effective policies and organizations [60]. Yu et al. applied the system dynamics to study the diffusion of public-sentiment, under dangerous chemical pollution cases, to discover and develop response strategies [61]. Using system dynamics and PCM methods, Xie et al. proposed the so-called “Parallel Evolution and Response Decision Framework of Public Sentiments” [62]. Jian et al. use system dynamics for simulation analysis to discover the dynamic changes in the number of cruise travel online customers in different life periods which considered Internet word-of-mouth and the information quality [63]; (b) CA simulations. The core of system dynamics is mathematical calculation. It concerns structural factors or variables, other than autonomous interactions of agents. This is relatively traditional and does not belong to Agent-Based Model. CA (Cellular Automata) is the elementary version of Agent-Based Modeling. Yao used CA simulations to explore the possible applications of online public-opinion events [64]. Zhang and Xiao proposed a CA model, considering neighbors, opinion leaders, and external influences, and group polarizations [65]. Wang et al. used CA models to analyze the evolutionary dynamics of public opinions, to uncover the steady state of the convergence speed of public opinions [66]. The pitfall is that individuals (agents) are not intelligent or smart and cannot make complex decisions; (c) intelligent-agent system simulations. Agent-Based Model is the most advanced method to simulate both natural and social systems by building a multi-agent system. For instances, Zhu and Hu studied the reversal law of public opinion, and verified it using ABM to simulate a case [67]. Yu et al. explored a crisis information release strategy of the mass media for controlling public panic stemming from emergency events, using four accidents of hazardous chemical leakage into rivers in China as case studies [68]. Tan et al. used the small world model to build agent-based model of online public-opinion propagations [69]. Jiang and Peng used the NetLogo software to analyze online public-opinion events, which is related to the H7N9 virus [70]. Huang et al. used ABM to explore the relationship between individual cognitive bias and choices or behaviors. The distribution of cognitive bias plays the key role in measuring the reversal probability, and ABM accurately describes and predicts the whole process of online events [71]. Jiang et al. use ABM model which explains the effects of clicking position and the number of items per online webpage on posting contributions [72]. Existing related Agent-Based Modeling has certain practical significance, but most stays at the quantitative description level. They have not seized the core mechanisms of individuals, such as the substitution effects and attention mechanism. Most Agent-Based Modeling aims at single or independent events, which do not focus on the life cycle trends. Hence, we take the core mechanisms into consideration, and build our agent model to investigate both single and coexisting online life cycles during this COVID-19 pandemic.

### Static settings of agents

**Fig. 2:**
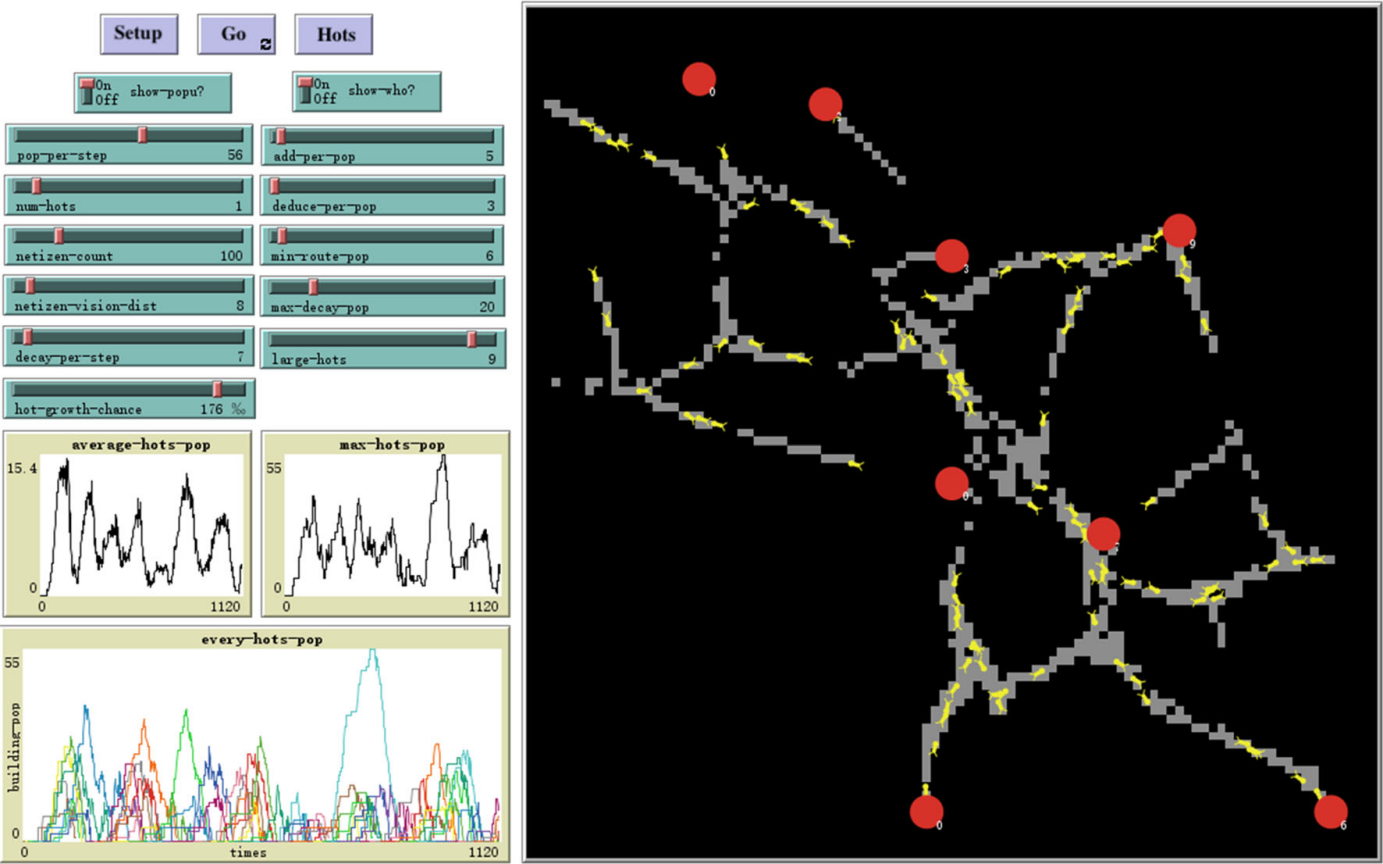
The modeling interface and simulation process. This is a simulation of online collective actions. The left part is related to command buttons and parameter settings (slides, monitors, and switches). The right part presents the dynamic process of simulations. The red solid circle refers to online events, chased or followed (visited) by Netizens in yellow. The gray patches refer to the pathways formed by moving agents (Netizens)

For online public-opinion events related to COVID-19, they are affected by many factors, such as subjects, objects, event, news media, and opinion leaders. According to the KISS principle (Keep It Simple and Stupid) [73], we build a multi-agent system to reproduce life cycles of online public-opinion events. As an intelligent software, NetLogo has both Patches (static agents) and Turtles (movable agents) [74]. For the settings of agents, Meng et al. used 200 media and 1400 netizens to simulate online public crisis events, and these numbers grew at each time [75]. Zhong set different shapes of agents (netizens) in NetLogo, to distinguish opinion leaders [76]. In these studies, agents cannot move. However, we focus on core attributions and interaction processes. In Fig. 2, we set a black square to represent the cyberspace with two classes of movable agents.

- Static settings of cyberspace: We use the static patches to represent the cyberspace world, which is complicated and complex in reality. To test its universality, representativeness, and robustness, we set the squared patch to construct the whole cyberspace where public opinions happen online. The world is a square, with the area of 101 × 101 = 10,201 (Patch^2^), and is not closed, but connected horizontally and vertically, which depicts the topological structure of permeability and connectivity [77]. The running time is measured by Ticks (hours), which automatically grows by one at each step. There are 2256 Ticks happened in 3 months. In general, online collective action events usually happen very quickly. If the model time (tick) is set to simulate everyday events, it will not reflect the characteristics of online collective action and is also inconsistent with the actual observation. Therefore, in this simulation, each Tick is calculated by hour for 24 h a day, that is, 24 ticks a day, to reflect the real situation.
- Settings of netizens: In our model, netizens are movable agents (meaning they can move freely), because netizens participate online topics following no restrictions. In 2019, China has 954 million netizens, but the increase rate is as slow as 1.6%. Therefore, we set the total number of netizens as a fixed value, because netizens maintain the same size within 3 months [78]. Therefore, the number of netizens is set to be within [100, 500]. Netizens have the attribution, “goals”, which make it for them to find the possible interesting target events to participate. Participation action includes webpage clicks, webpage visiting, comments, interactions, and so on. As movable agents, the Hots refers to online collective actions or public-opinion events. Previous researchers regard them as static and passive aggregated results, but we take them as the macro-level process formed by movable and autonomous agents. They take places suddenly and randomly in cyberspace, but their number is pre-defined, because the attention of all citizens is limited.
- Settings of Hots: Thus, we set the number of Hots to be within [1, 10], which can be adjustable under different simulations. The key attribution of Hots is “Pop”, which indicates the daily visiting frequency (popularity) of certain simulated events. Therefore, the “Pop” is dynamic, and the Hots will die if the “Pop” value is zero. When old events die, new events will be generated and visited by Netizens. There are certain probability levels (hot-growth-chance) for new events to be created. For each visit to each Hot by netizen, a random increase in popular value (add-per-pop) is added. Within the range of [1, 100], the value (add-per-pop) is adjustable. For different Netizens, the increase of popularity may be heterogeneous. In big data era and social media time, strangers online can form communities, shape public opinions, and launch online cases [79]. The boundary between individuals and opinion leaders is blurry.
- Mechanism designs of life cycle dynamics. Based on these considerations, we introduce the random weight coefficient ${w}_{it}\in [0, 1]$ to manifest the contribution degrees for different individuals or agents, and to distinguish the opinion leaders from common netizens. This weight coefficients are random for all agents, and we do not specifically differentiate subtypes of Netizens, which is in accordance with the reality. Based on reasonable mechanism designs of individual behaviors, we set them up to present the life cycle trends. As the simulation process goes on, the popularity value of each Hot (event) $k$, at each time $t$, can be dynamically calculated. When the popularity value (${\mathrm{Pop}}_{kt}$) declines to zero, the corresponding events with ${\mathrm{Pop}}_{kt}=0$ will die. This regularity can be applied to both old and new events. The multi-event system constantly goes on until the ending conditions are satisfied, and related parameters and interpretations can be seen in Table 2.

**Table 2:** Main parameters and illustrations

Parameters	Meanings	Values
Add-per-pop	The added popularity value per visit of netizen for Hots	1–100
Deduce-per-pop	The deduced popularity values per Ticks for Hots	1–100
Hot-growth-chance	The generation probability of Hots	1–100‰
Netizen-count	The total number of netizens in model	1–500
Num-hots	The original number of Hots in the model	1–10
Large-hots	The largest number of Hots in the model	1–10
Netizen-vision-dist	Finding the path range of netizens in the vicinity	1–100
Pop-per-step	The added path popularity values for the patch of netizens	1–100
Decay-per-step	The recessing rate of patch’s path popularity values	1–100%
Max-decay-pop	The threshold value of the patch’s recessing path popularity values	1–100
Min-route-pop	The threshold value of patch’s path popularity values	1–100

### Behavior rules and dynamic settings

According to the theory of attention allocation, the running process of simulations is as follows: (a) initialization and agent settings. Internet world, Netizens, and Hots are generated initially in Netlogo. The initial locations of Hots are random and the number of Hots is as set. Due to the limited attention of Netizens and the Head law effect, there are not too many online events within certain time periods. Therefore, only a certain small number of Hots are generated initially. Then, new Hots are generated when old ones die, and a fixed number of Hots will be maintained. The number of netizens is fixed for each simulation. After the simulation begins, Netizens move around to find their goals (Hots), and pursue them; (b) netizens will search targets and visit Hots. There are two modes for Netizens to chase and visit Hots. The first is random visiting mode, which indicates the disordered behaviors and random visiting of Netizens. The second is swarm intelligent mode, where Netzines interact with each other. Netizens will prefer to follow the pathways of neighbors, because in reality, people tends to follow and read postings and sharing of their friends or acquaintances. The interplay between Netizens is real, because the attitudes and behaviors of individuals are affected by others and the social environment [80]. Each mode has implications in reality, and we combine these two modes. They first follows other Netizens within a certain distance (netizen-vision-dist), and then, they have to walk and search for Hots randomly if there is no neighbor within this distance (netizen-vision-dist); and (c) Stopping visiting old events and running for new ones. For each Hot, the visiting times of Netizens is limited or fixed, which coincides with people’s limited attention law. Hence, active Hots will die because of the declined visiting after the peak, and netizen will run for new Hots to visit. In reality, diminishing marginal effects are common phenomena in human societies. Netizens are inclined to get bored with the multiple exposures of the same news or public opinions; therefore, their visiting times tend to be limited. After the COVID-19 outbreak, there are many related online collective actions. Due to government controlling measures (social distancing and home quarantine), people have more time to participate in the event online.

### Mathematical expressions of life cycle processes

In the system, old Hots gradually become weaker, and finally disappear, and at the same time, new Hots are visited by Netizens randomly, which is an automatic process. We use $i$ to refer to an arbitrary Netizen, and $k$ refer to single an arbitrary Hot event. This includes: (a) a macro-level system. For each Hot event $k$, the life cycle function (${\mathrm{Pop}}_{kt}$) stands for dynamic popularity values at the time $t$. The duration *T* is the maximum time, and when $t\in [0, T]$, ${\mathrm{Pop}}_{kt}$ characterizes the dynamic life cycle process*.* When ${\mathrm{Pop}}_{kt}>0$, this event $k$ is accessible and can be visited by netizens. When ${\mathrm{Pop}}_{kt}=0$, this event $k$ vanishes or dies, and it cannot be accessed or visited by netizens any more. In Eq. (1), the term ${\mathrm{POP}}_{Kt}$ aggregates all life cycle functions (${\mathrm{Pop}}_{kt}$) within this system, and represents the total amount of popularity or total online attention at the time $t$. We use Eq. (2) (derivatived form) and Eq. (3) (differential form) to capture the mathematical dynamics; (b) the life cycle dynamics. For single event $k$, the dynamic popularity variation of the life cycle function ${\mathrm{Pop}}_{kt}$ can be calculated as ${\Delta }_{k}^{t}$ minus the fixed loss $C$. There are four logical processes for this variation ${\Delta }_{k}^{t}$. The first one is the 0–1 logical value ${G}_{i\to k}^{t}$, which implies that the Netizen $i$ does take Hot event $k$ as its target or goal, at time $t$; the second is the 0–1 logical value ${V}_{i\to k}^{t}$, which indicates whether the agent $i$ is able to visit this Hot event $k$. If the total number of visiting $k$ has been satisfied, it cannot be visited, despite that $k$ has been taken as the goal of agent $i$. Thus, if event $k$ perishes or its maximal visiting time is reached, visiting $k$ becomes impossible. If this happens, agent $i$ will change its goal or target at the next step ($t+1$)*.* This mechanism guarantees the life cycle phenomena of the Hots. The third is a continuous variable ${A}_{ik}^{t}$, which refers to how much this visiting ($i\to k$) add values to current popularity of Hot $k$. For different settings, ${A}_{ik}^{t}$ can be heterogeneous. The fourth term is the continuous variable ${w}_{ik}^{t}$, which measures the weightings of different agents. For some agents, visiting will add more value to Hots; and (c) natural recession and cooling mechanism. The cooling or recession mechanism is the key to generate the vanishing stage and present the whole life cycle trends. In Eqs. (2) and  (3), the fixed loss *C* is natural and inevitable. Thus, the popularity value of Hots is depleted by the cost *C*, under a certain probability level (deduce-per-pop) at each running time. For each simulation, the values were taken randomly within the range of 1–100. This is to reflect the phenomena of natural cooling and inevitable recession of real online events. In Eq. (3), the dynamic popularity value of an event is the total popularity value at a previous moment pluses the added popularity value at this moment and reduces the fixed loss of *C*

1 $${\mathrm{POP}}_{Kt}=\sum_{k=1}^{k=K}{\mathrm{Pop}}_{kt}={\int }_{k=1}^{k=K}{\mathrm{Pop}}_{kt},$$

2 $$\frac{d{\mathrm{Pop}}_{k}}{dt}={\Delta }_{k}^{t}-C=\sum_{i=1}^{i=N}{G}_{i\to k}^{t}{B}_{i\to k}^{t}{w}_{ik}^{t}{A}_{ik}^{t}-C={\int }_{i=1}^{i=N}{G}_{i\to k}^{t}{B}_{i\to k}^{t}{w}_{ik}^{t}{A}_{ik}^{t}-C,$$

3 $${\mathrm{Pop}}_{kt}={\mathrm{Pop}}_{k,t-1}+\sum_{i=1}^{i=N}{G}_{i\to k}^{t}\bullet {B}_{i\to k}^{t}\bullet {w}_{ik}^{t}\bullet {A}_{ik}^{t}-C.$$

## Optimal solution and simulation outcomes

### The optimal combination of parameters

According to dynamic life cycle processes of real events, we found the optimal (best-fitting) combination of parameters, which is the optimal solution of our Agent-Based Modeling and simulations. We traversed all related parameters to find the optimal combinations. Big data characteristics of real events are taken to form the target function ${f}_{\mathrm{sim}}\left(\bullet \right)$, which will be fitted by simulation outcomes ${f}_{\mathrm{real}}\left(\bullet \right)$. Within the set {1, 2, 3, …, 500}, one Netizen (netizen-count) to 500 hundred Netizens are traversed; the visiting increase (add-per-pop increases) is within the set {1, 2, 3, …, 100}; the cooling effect (deduce-per-pop) is from the set {1, 2, 3, …, 100}; the largest number of Hots (large-hots) is from {1, 2, 3, …, 10}; the “hot-growth-chance for new events” grows from 1 to 200‰; the “netizen-vision-dist” takes values from {1, 2, 3, …, 100}. Each unique combination of parameters produced one simulation result, and we used 10,000 simulations for parameter traverse. Each simulation was repeated for ten times, and the average values were taken as the robust results, to obtain ${f}_{\mathrm{sim}}\left(\bullet \right)$. Hence, parameter traverse was done for 100,000 times. In Eq. (4), we calculated the difference ($\Delta$) between simulations and real big data, using the calculations of MSE

4 $$\mathrm{Parameters}\left(*\right)=\mathrm{ArgMin}\left(\Delta \right)=\mathrm{ArgMin}\left[{f}_{\mathrm{sim}}\left(\bullet \right)-{f}_{\mathrm{real}}\left(\bullet \right)\right].$$

When the difference ($\Delta$) is minimal, the optimal parameter combination, $\mathrm{Parameters}\left(*\right)$, can be obtained to achieve the maximum degree of fitting or matching. Based on 100,000 simulations, the optimal solution $\mathrm{Parameters}\left(*\right)$ fit our big data objects best. The closer the number of simulation event sets is to the number of real event sets, the more likely it is to match the optimal solution. Therefore, we consider the number of priority matching event sets to predict the possible optimal solution. In the process of the simulation parameters traverse, the parameters with similar number of event sets are selected as the optimal solution, and then, multi-dimensional matching is carried out to verify that the optimal solution meets the requirements. (We have taken three matching criteria into consideration. The first criterion is event number matching, the second criterion is the matching of event life distribution, the third criterion is life cycle curve matching.) The optimal solution is one unique combination of parameters that are listed in Table 3. The number of Netizens is 100 (netizen-count = 100); each visit adds five popular values (add-per-pop = 5); the cooling effect or the Cost should be 3, at each time (deduce-per-pop = 3); the probability for new Hots to be generated (hot-growth-chance) should be 176‰; the system should maintains nine events during the whole simulations (large-hots = 9); and the distance for Netizens to search neighbors should be eight patches (netizen-vision-dist = 8).

**Table 3:** The parameter values of the optimal solution

Netizen-count	100	Pop-per-step	56
Add-per-pop	5	Decay-per-step	7
Deduce-per-pop	3	Min-route-pop	6
Hot-growth-chance	176‰	Netizen-vision-dist	8
Large-hots	9	Max-decay-pop	20

The comparison between real big data ${f}_{\mathrm{sim}}\left(\bullet \right)$ and simulations ${f}_{\mathrm{sim}}\left(\bullet \right)$ is based on big data trends and features, such as morphology and data comparisons. For a single event, there are curve shapes, peak heights, peak times, and life durations. For multiple events, there are the number of emergences (happened events), trend comparison, peak interval, substitution interval, and peak difference. For comparing distributions of big data and simulations, we applied both absolute and relative distributions. Under $\mathrm{Parameters}\left(*\right)$ that have achieved the best fitness, we ran simulations for 10 extra times to show both effectiveness and robustness of the optimal solution. Based on the optimal solution simulation outcomes (*N* = 10), we obtained the robust distributions of variables and outcomes.

### Same number of events during same time

**Fig. 3:**
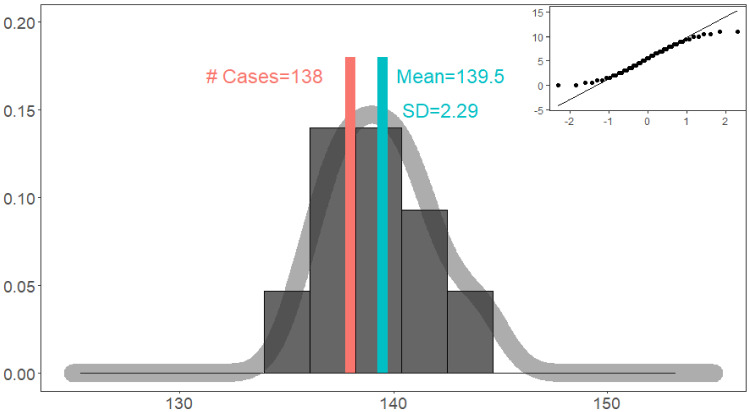
The quantity matching of simulations. The mean is 139.5, close to that of 138 real events. The *Q*–*Q* plot indicates that the number of events follows the normal distribution for regular numbers of events. This indicates that repeated simulations match real target events

Figure 3 indicates that repeated simulations (*N* = 10) perfectly match real target events. We check three aspects: (a) accuracy. We have 138 real online events. For simulations, the mean is 139.5 online events. The percentage of bias (2.8) is merely less than 2% of the mean value. Moreover, the SD (standard deviation) is merely 2.29, and the bias is within one SD, which implies that they have no statistically significant differences; (b) robustness. Figure 3 indicates a good centrality distribution tendency. It implies that the action mechanism is reasonable and robust. There is no non-linearity, such as sudden changes or phase changes, and the simulation results are close to the average levels; and (c) predictability. In addition to the centrality, the symmetry of the distribution is fair. The *Q*–*Q* normal plot indicates that the number of events follows the normal distributions with a higher probability. We are able to obtain the complete information of the probability density function for predictions by calculating Empirical Density Function with Mean and SD values.

### Macroscopic matching of life cycle trajectories

**Fig. 4:**
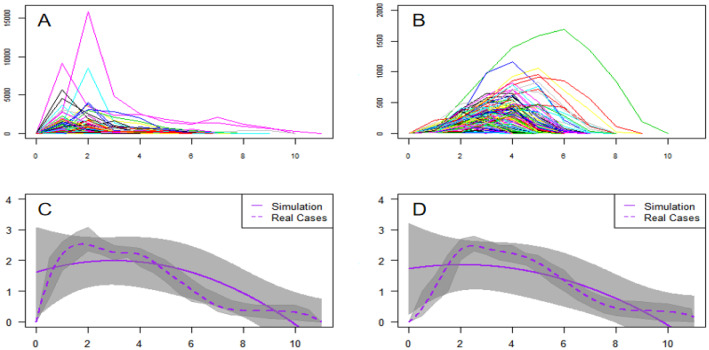
Life cycle characteristics and optimal solution simulations. Panel **A** visualizes the life cycle trends of real events, and Panel **B** shows simulated life cycles. Panel **C** compare outcomes of simulations (each 24 h) with real events, and Panel **D** compares simulations (each 48 h) with real events

Because online events show robust life cycles, optimal solution simulations should match this pattern. At the horizontal *X-*axis, the duration or lifespan is the key indicator of both real and simulated life cycles. Other than natural days, the life cycle is from the 1st, 2nd, and up to the last day. Figure 4A shows the life cycle curves of real COVID-19 online events. Each event can be represented by a two-dimension array ($t,{y}_{\mathrm{real}}$), which records the whole dynamics of popularity ${y}_{\mathrm{real}}$. Similarly, Fig. 4B shows simulated life cycle curves, and each event is denoted by ($t,{y}_{\mathrm{sim}}$). For real-life cycles (events), the durations are usually less than 12 days, and the peaks can be reached at early stages. To compare real and simulated events, we calculated and visualized the life cycle trends. The average popularity can be calculated according to the ranked days (the 1st, 2nd, and 3rd day). Thus, we obtained aggerated real-life cycle function ($t,{\widehat{y}}_{\mathrm{real}}$) and simulated function ($t,{\widehat{y}}_{\mathrm{sim}}$) and compared them in Fig. 4C, D. We took logarithm values before we did the calculations. Figure 4C (1-day basis calculations) and Fig. 4D (two-day basis calculations) compare real (dotted line) and simulated life cycles (solid line). The real big data are observed at one time, and the smoothness cannot be guaranteed. Results of ten paralleled simulations are more ideal and smoother, with upper and lower bounds. Comparing Fig. 4C with Fig. 4D, the matching is better on a 2-day basis.

### Matching of life cycle durations

**Fig. 5:**
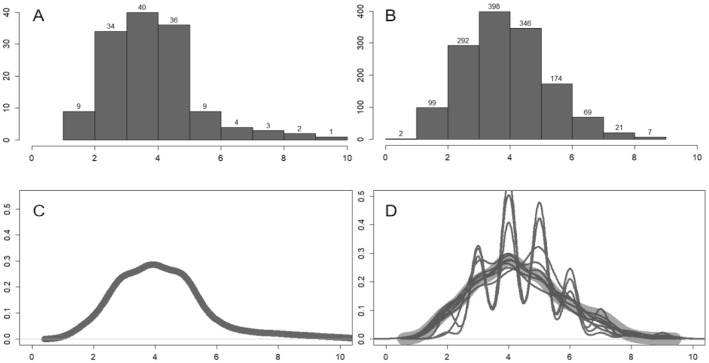
The distribution matching of life cycle spans (durations). Panel **A** shows the distribution of real durations (*N* = 138), while Panel **B** indicates the distribution of simulated durations. In Panel **C**, we calculated kernel density function of 138 real events, and we visualized the kernel density function of simulations in Panel **D**. The overall trend and the local trajectory values are both precisely matched

We have: (a) the duration matching. The Chi-square test is used to compare the outcomes of simulated and real durations. *H*_0_ is that they are equal, while the *H*_1_ is unequal. The simulation data are group One, and real big data is group Two. It seems that ${\chi }^{2}$= 0.046 and *P* value is 0.831 > 0.05. Thus, we accept *H*_0_ and there is no statistical difference between real and simulated data. The performance of the optimal solution is robust and excellent; (b) discrete distribution matching. The distributive matching is calculated in discrete and continuous forms. Most real durations are within [2, 10] days, with a high-density interval of [2, 6] days. We visualized and compared the discrete distributions for real big data in Fig. 5A and simulated outcomes in Fig. 5B. It seems that simulations match real events very well. First, their histograms are similar in shapes and heights. Second, the distributions of extreme values are matched for 2 and 10 days, respectively. Third, the distributions of middle high-density values are matched from 2 to 6 days. Fourth, the distribution ranks of detailed durations are the same for both, such as 4 days > 5 days > 3 days; and (c) continuous distribution matching. Time can be divided infinitely. If converted to 24 h a day, there are 2.5, 2.75, and 8.025 days, as well. Corresponding to discrete distributions of Fig. 5A, B, we further check the continuous distributions (Kernel Density Function, KDF) of real and simulate durations. It indicates that they are perfectly matched. In Fig. 5C, it shows the continuous distributions of real durations. Figure 5D shows ten distributions for ten simulations, and the overall distribution is the same as in Fig. 5C. It implies that our Agent-Based Modeling and simulations have captured the core mechanism of the reality.

### Peak and shape matchings of life cycles

**Fig. 6:**
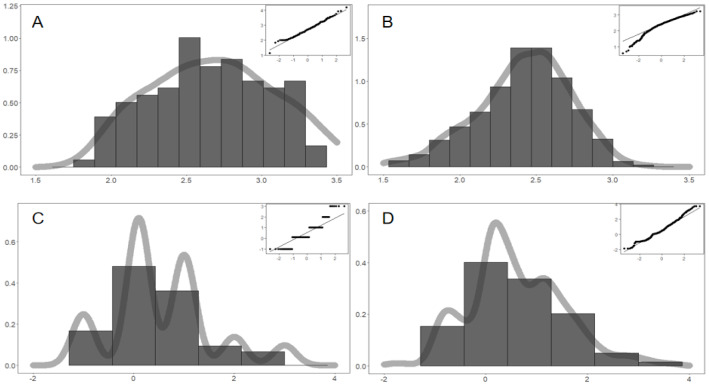
Matching of peak height and peak intervals. We compared the distributions of real peaks and simulated peaks. It indicates that there is no statistically significant difference between simulated and real values; therefore, the substitution mechanism exists

**Fig. 7:**
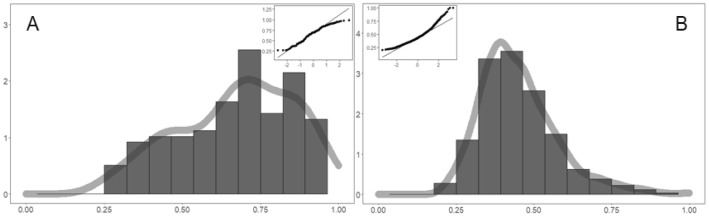
The matching of relative peaks. We checked two distributions of relative ratios (peak/duration), for real events (Panel **A**) and simulations (Panel **B**). This indicates again that simulations fit well

Figure 4 and 5 are verified the validity and robustness of our simulations. For life cycle functions, durations are macroscopic results. We continue to examine the morphological characteristics, such as shape and peaks. If the duration (lifespan) is the key index on the *X*-axis, then peak is ought to be the key index in the *Y*-axis. Hence, the life cycle function can be characterized by peak (*Y*-axis) and duration (*X*-axis) [81]. Peak and duration are of equally importance, and they jointly determine the life cycle trends. We have the following findings:

- Matching of absolute peak values: To a large extent, the peak determines whether online events have enough impacts or power to achieve goals, such as attracting social attention, exerting public-opinion pressure, and promoting problem solving. The peak model (2016) has been proposed to capture human group behaviors [82], and here, we focus on pandemic-related online collective actions. The simulated and real peaks (logarithm) are examined from two aspects. The first step is to compare means. The distribution of real peaks is shown in Fig. 6A, with the mean of − 2.74 and SD (standard deviation) of 0.50. The simulated peak distribution is shown in Fig. 6B, with Mean = − 2.37 and SD = 0.41. There is no statistically significant difference between them, and perfect matching can be statistically supported. The second is to check the distribution matching. The real peak distribution in Fig. 6A and the simulated peak distribution in Figure B are found to be well aligned. It is found that the probabilities of the two are close to normal distribution through *Q*–*Q* normal plots. It indicates that both ranges of simulated and real peaks are within [1.5, 3.5], which is highly consistent. Therefore, given the same *X*-axis range, there is no statistically significant difference between simulated and real peak values, which fully shows that our simulations match the real situations very well.

(b) Substitution effects and peak interval matching: For real cyberspace, multiple events happen at the same time (day), which constructs a whole system emergence. Every event has a life cycle, the core feature of which is the peaks indicating the greatest influence and social impact. In this dynamic system, there are multiple life cycle curves. Since we have mentioned the substitution mechanism for coexisting events in reality, our optimal solution should match real big data trends. If this substitution mechanism holds, the nearest neighbor should have the greatest substitution possibility, because they are too close to avoid competing for the attention of Netizens. There is no existing method to measure this substitution effect, but it is feasible to use peak-day gaps or differences. The real big data in Fig. 6C show the distribution of peak-day gaps between two adjacent peaks (events). Our Mean = 0.60 and SD = 1.98, which indicates that it takes about 0.6 days for new event to substitute previous old one. The distribution of simulated peak gaps in Fig. 6D indicates that Mean = 0.64 and SD = 0.92. It takes about 0.64 days for old events to be replaced by new events. The two means are close to each other, and they have no significant difference considering two standard deviations. In addition, the shapes of the two distributions are similar as well. For instances, the highest density day are both at the 1st day, and they both have the right tails. It suggests that two distributions are both close to the normal distribution. Hence, the substitution mechanism really exists, and our simulation reflects and back-calculates real events very well.
(c) Relative Peaks Matching (Peak/Total): We further check the relative peak, which largely determines the shape of life cycle curves. “Total” represents the total amount of participation. The Peak/Total can be deemed as the relative strength of peaks. The greater the ratio (Peak/Total) is, the more abrupt and sudden the event will be, and the greater impacts the peaks will have. Smaller ratio (Peak/Total) refers to more gentle and smoother life cycle processes. For real big data in Fig. 7A, the Mean = 0.66 and the SD = 0.18. It suggests that the peak-day participation accounts for roughly 66% of the total participation in reality. For simulations in Fig. 7B, the Mean = 0.45 and the SD = 0.13. Two distributions are largely overlapped, and two means have no statistical difference. Besides, two scopes of real ratios (from 0.25 to 1) and simulations (from 0.2 to 1) are largely overlapped, which indicates again that simulations fit well.

### Matching of micro-level behaviors

Previous verifications are based on macro-level indicators or life cycle shapes, and we further checked the outcomes of individual behaviors, for both real big data and simulations. The life cycle function ${\mathrm{Pop}}_{it}$ is calculated on a daily basis (24 h), and we also calculated them on a multiple-day basis, such as 3, 5, and 7 days. It seems that the outcomes of the real big data and simulation are matched, as well.

**Fig. 8:**
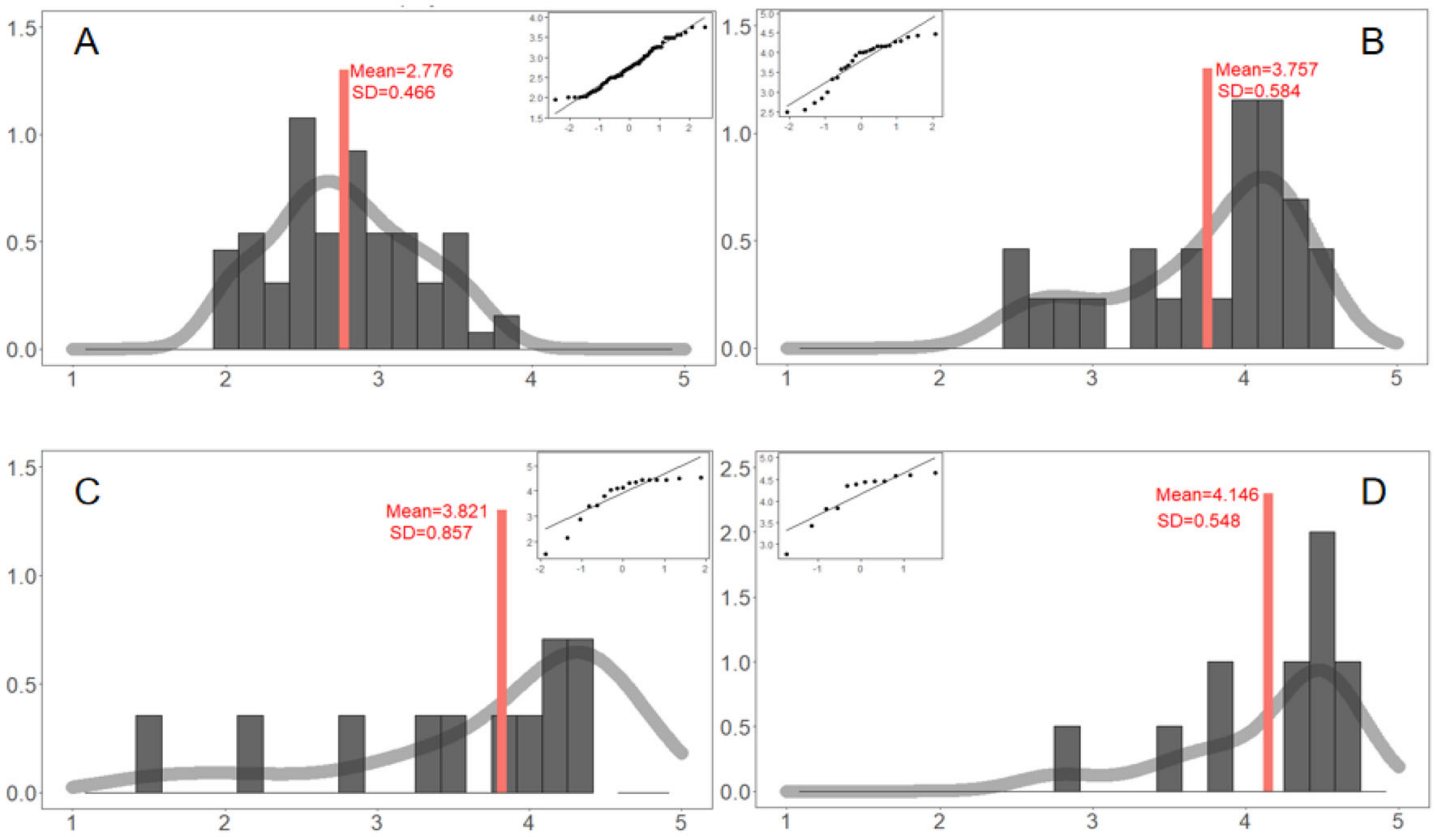
Real distributions of 1-3-5-7-day participations (logarithm). For each panel, we plotted the distribution and checked the normality. Distributions of real big data should be matched by the optimal solution

**Fig. 9:**
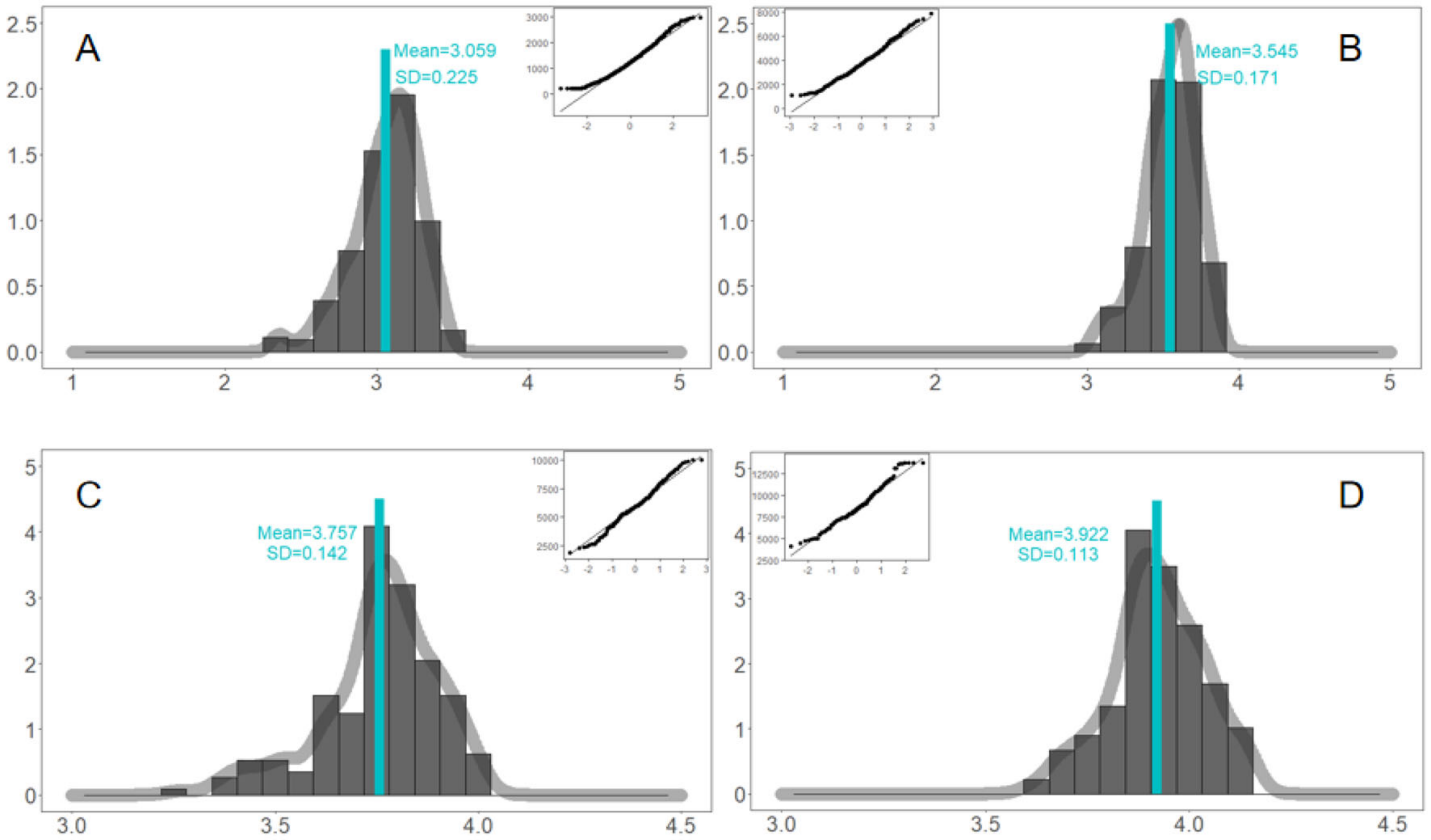
Simulated distributions of 1-3-5-7-day participations (logarithm). For each panel, we plotted the distribution and checked the normality in the *Q*–*Q* plot (subfigure). The simulation well matches the real data

- Daily participations are matched. For real big data in Fig. 8A, the Mean = 2.776 and the SD = 0.466 for the daily participation (logarithm). The distribution is good bell curve, and the *Q*–*Q* plot indicates a normal distribution. For simulations in Fig. 9A, the Mean = 3.059 and the SD = 0.225 for the daily participation (logarithm). The distribution is also close to a perfect bell curve, and *Q*–*Q* plot suggests a normal distribution for middle-range values. The real range of [2, 3.8] can fully cover the simulated range [2.3, 3.5], which is narrower and therefore more robust. Hence, simulation results largely match or coincide with the real big data, and there are no significant differences between them.
- 3-day participations are matched. For real big data in Fig. 8B, the Mean = 3.757 and the SD = 0.584 for the distribution of 3-day participation (logarithm). The distribution is skewed, but still shows certain symmetrical characteristics when extreme values are removed. The *Q*–*Q* plot shows that it is close to normal distribution except for extreme values. For simulation values in Fig. 9B, the distribution of 3-day participations seems to be perfectly symmetric and bell-curved, with the Mean = 3.543 and the SD = 0.171. The *Q*–*Q* plot supports the normal distribution as well. It suggests that both real and simulated 3-day participations are perfectly matched, because they have no significant differences statistically. The two mean values are close to each other, and two distributions are largely overlapped. Besides, real value range [2.4, 4.5] can fully cover simulated values range [3, 3.85], which is narrower and more robust.

(c) 5-day participations are matched. For the real big data in Fig. 8C, the Mean = 3.821 and the SD = 0.857 for the distribution of 5-day participation (logarithm). The distribution is more diverse, with some more values near a standard deviation of the mean and a smaller discontinuous range. The middle non-extreme value is skewed to the left, and there is a certain extreme value on the left. *Q*–*Q* test shows that after removing the extreme values; the residual data approximately follow the normal distribution. Figure 9C shows the number of the 5-day posts, and the logarithm of simulation Mean = 3.757 and the SD = 0.142. It is symmetric and neutral, and generally follows the normal distribution. *Q*–*Q* test shows that most of the data are on the straight lines. The range of real values are [1.4, 4.5], and the simulation data are concentrated more in [3.5, 4], which is completely included by the former, and there is no significant difference between these two mean values. The convergence validity of the simulation is higher.
(d) 7-day participations are matched. Figure 8D shows the 7-day real-life participation volume (logarithm), with the Mean = 4.146 and SD = 0.548. There are also many discontinuous values from 3 to 4.8. The middle-range (non-extreme) values are skewed to the left, and more extreme values are on the left. The *Q*–*Q* test shows that it may follow the normal distribution if extreme values are removed. As in Fig. 9D, the Mean = 3.922 and the SD = 0.113 for the simulated 7-day participations. Comparing two mean values, it seems that no statistical differences exist between them. For the value ranges, it is [2.6, 4.8] for real events and [3.6, 4.2] for simulations. The range of simulations is completely within the range of real (target) events. Hence, the outcomes of simulations are more narrowed and focused, and the convergence efficiency of simulations is much higher. It reinforces the convergence validity and robustness of our ABM modeling, simulations, and even predictions.

## Conclusions and discussion

Our model unveils key microscopic mechanisms of human collective action, and therefore facilitates high-precision simulations and life cycle process reproductions of pandemic-related online collective actions.

- Rules exist for the emergence of online human actions at macro-level. Online collective action is a typical human behavior, which has the life cycle law. In cyberspace, a large number of events can be witnessed every day. In our work, all events have life cycle features, and there are no exceptions so far. We first checked the rules and regulations of single life cycles. Big data mining also shows that most lifespans are within 12 days, which is the 2-week rule. For most events, they have the lifespan of 7 and 8 days (one). During the pandemic, the most related events have a lifespan of 3–5 days. Then, we investigated the interactions of coexisting or multiple life cycles (events). Obviously, new events will definitely influence (shorten) the life cycle process of old events, which is the substitution effect. In other words, the emerging of new events has negative effects on old ones.
- Features of COVID-19 online collective actions are obtained. Based on big data mining, we obtained the following characteristics for online collective actions. First, the number of public opinions (participation) increased sharply, and 138 online events were found within merely 3 months. Second, the durations of the events are short, and it is hard to witness very long durations. The percentage of online events within 3–5 days is 79.71%, which indicates the features of rapid outbreaks and extinctions of events. This is different from other online events. Third, the substitution effect is obvious and strong, as we see many pairs of peaks that are close to each other. Finally, domestic and international public opinions are intertwined. In the past, most collective actions are local and isolated events with limited or constrained influences. Under the big data era, COVID-19 online collect actions can easily penetrate the world and have global impacts. Big data features of 138 events provide target objectives of our modeling and simulations.
- The micro-level actions hold the macro-level phenomena. Macro emergence action needs micro-level action support. Abott called it “macro effect originated from micro level” [83]. The so-called “emergence” refers to macro-level phenomenon caused by specific rules of micro-level actions. In other words, the macro-level evolutionary dynamics are shaped by behaviors of automatous agents who act and make adaptive adjustments. Thus, online collective actions should be supported by micro-actions of Netizens. If our model holds, the outcomes of simulations should match the real big data trends. The application of ABM also solves the “repeatability crisis” [84], which is a long-lasting pitfall of social sciences. We created two kinds of agents (netizens and events), who act under the action mechanisms, such as the cooling, substitution, and attention mechanisms. The fitness to reality is good. The whole process is controllable and repeatable.
- Mechanisms of cooling, substitution, and attention shift determine the life cycles. Online collective actions will have no life cycles (grows all the time) if there is no negative mechanism. Thus, the key to solve the life cycle process is the negative mechanisms, which are cooling, substitution, and attention-shift mechanisms applied here. The cooling effect is caused by the diminishing marginal utility, which is universal in human behaviors. On the Internet, the subjective utilities of netizens will surely decrease as the time goes. Therefore, this cooling process is natural and inevitable. Besides of the cooling mechanism, the substitution effect accelerates the vanishing of life cycles. As the total attention of Netizens is limited, the attention shift, from old events to new ones, will definitely make old life cycles end earlier. The total attention limitation is the roots of both cooling and substitution effects. During the pandemic, people have much more time to browse online information; therefore, the total attention amount is much higher than pervious times, but it is still fixed or limited.
- Agent-Based Modeling has critical advantages. Agent-Based Modeling reveals macro-level emergence patterns or features through the mechanism design of micro-level actions. The advantage is that it can represent the dynamic process. Quantitative model and even big data mining are used to discover knowledge from the static data, which is one snapshot of the world. These models cannot represent the complex and dynamic process of interdisciplinary studies. Agent-Based Modeling and simulations can not only explain the static data of social phenomena, but also the dynamic process. The interpretations, knowledge, and predictions of ABM will be dynamic, continuous and procedural. It vividly presents the interaction process of Netizens, and records the real-time status of the whole system. For the simulations of Agent-Based Modeling, its reasoning logic and argument process can be visualized; therefore, what the readers see is what the authors propose.
- The optimal solution of simulations is robust, adaptive, and conditional. According to the big data characteristics of the target real events, we use multiple simulations to find the optimal combination of parameters. First, this optimal solution should be robust. To avoid randomness and disturbances, the optimal solution simulations is repeated by ten times. The mean value and distribution matching degrees have been achieved. Second, the optimal solution of the simulations should be adaptive. The 138 online events are only an example to prove the robustness of life cycle model. A new set of optimal parameters will be found if we change the target events. The matching degrees will be also achieved, which fully reflects the adaptability of our model and parameter settings. Third, optimal solutions should be conditional. If target events change, optimal solutions will be different. Robustness, conditionality, and adaptability are not only attributions, but also matching indicators of the simulations.
- Public policy implications: For malicious online collective actions (rumor spreading), the government should overcome their negative social impacts. First, malicious online events can be contained by the substitution mechanism. This is a regular operation, which includes releasing or generating other hot events to dilute present events. Our model indicates that the semantic distance matters, and new events with similar contents and features will have strong substitution effects. Same theme will be concerned by the same group of netizens, and new events can accurately attract certain groups. Second, malicious online events can be undermined by the attention-shift mechanism. Through this mechanism design, the attention (popularity) of online events will be reduced when new events happen. These practical responsive strategies can be supported by our life cycle modeling.
